# Quantitative assessments and clinical outcomes in HER2 equivocal 2018 ASCO/CAP ISH group 4 breast cancer

**DOI:** 10.1038/s41523-019-0122-x

**Published:** 2019-08-29

**Authors:** Swati Gupta, Veronique Neumeister, John McGuire, Yan S. Song, Balazs Acs, Kenneth Ho, Jodi Weidler, Wendy Wong, Brian Rhees, Michael Bates, David L. Rimm, Veerle Bossuyt

**Affiliations:** 1 Department of Pathology, Yale University School of Medicine, New Haven, CT USA; 2 Indivumed Inc, Frederick, MD USA; 3 Department of Oncology and Pathology, Karolinska Institute, Stockholm, Sweden; 4 Division of Oncology Research and Development, Cepheid, Sunnyvale, CA USA; 5 Medical and Scientific Affairs and Strategy, Oncology, Cepheid, Sunnyvale, CA USA; 6 Massachusetts General Hospital, Boston, MA USA

**Keywords:** Cancer, Breast cancer

## Abstract

We quantified human epidermal growth factor receptor 2 (HER2) RNA and protein expression in 2018 American Society of Clinical Oncology/College of American Pathologists (ASCO/CAP) in situ hybridization (ISH) group 4 (*HER2/centromeric probe 17* (*CEP17*) ratio <2.0, average *HER2* copy number ≥4.0 and <6.0, and 2013 ASCO/CAP ISH equivocal) breast cancers. Breast cancers in 2018 ASCO/CAP ISH group 4 between 2014 and 2017 were identified from the Yale archives. Sixty-three patients (34 with HER2 immunohistochemistry (IHC) 0/1+ and 29 with HER2 IHC 2+) were included. We compared patient characteristics, systemic treatments, and outcomes. We assessed HER2 by real-time quantitative reverse transcription polymerase chain reaction (RT-qPCR) and quantitative immunofluorescence (QIF). Among ISH group 4 cancers, higher *HER2* mRNA (*P* < 0.0001) but similar HER2 protein levels were observed in IHC 2+ compared to IHC 0/1+ cancers. The distribution of RT-qPCR and QIF scores were independent of fluorescence in situ hybridization (FISH) ratio/copy number. Concordance between HER2 RT-qPCR and QIF was 69.8% (*r* = 0.52). Among 29 patients with IHC2+ results, 16 were HER2 positive by RT-qPCR and 12 were HER2 positive by QIF. Systemic treatment, recurrence, and survival outcomes were comparable among ISH group 4 cancers regardless of IHC 0/1+ or 2+ results. ISH group 4 cancers appear to form a distinct group with intermediate levels of RNA/protein expression, close to positive/negative cut points. Therefore, adjudication into positive or negative categories may not be meaningful. Our results support the 2018 ASCO**/**CAP recommendation to refrain from routine additional testing of these samples. Additional outcome information after trastuzumab treatment for patients in this special group might help to guide treatment decisions in these patients.

## Introduction

Breast cancer can be classified into two major clinically important groups by immunohistochemistry (IHC): human epidermal growth factor receptor 2 (HER2) positive (IHC 3+) and HER2 negative (IHC 0/1+). However, by IHC, up to 18% of all newly diagnosed breast cancers fall into a third category defined as HER2 equivocal (IHC 2+). Patients with HER2 positive tumors (IHC 3+) are eligible for anti-HER2 therapy. According to American Society of Clinical Oncology and the College of American Pathologists (ASCO/CAP) guidelines, equivocal IHC results (IHC 2+) should be reflex in situ hybridization (ISH) tested on either the same or an alternative specimen.^1^ Patients with ISH HER2/centromeric probe 17 (CEP17) ratio ≥2.0 or with HER2 copy number >6.0 are eligible for anti-HER2 therapy. Reflex testing resolves most cases; however, a subset remains difficult to classify by ISH when ISH *HER2/CEP17* ratio is <2.0 with *HER2* copy number 4.0–6.0 (ISH group 4 in the 2018 ASCO/CAP guidelines).^1,2^ It is unclear whether these patients benefit from anti-HER2 therapy. Several methods have been proposed to resolve such uncertainty. Real-time quantitative reverse transcription polymerase chain reaction (RT-qPCR)-based tests have been proposed, because *HER2* gene amplification is associated with *HER2* transcript overexpression.^3–5^ *HER2* fluorescence in situ hybridization (FISH) with alternative chromosome 17 probes has been proposed to reclassify a subset of these cases as positive based on *HER2/CHR17* probe ratio.^6,7^ Given the complex chromosome 17 rearrangements that can be seen in breast cancer *HER2/CHR17*, probe ratios based on alternative chromosome 17 probes may also not be a reliable indicator of the true gene amplification status. The use of alternative probes is discouraged in the 2018 ASCO/CAP guidelines in view of the absence of outcome data.^1^ Recently, genomic profiling identified a small subset of HER2-enriched carcinomas among ISH group 4 carcinomas.^8^ Adjudication by these methods has not been associated with outcome after trastuzumab treatment. Therefore, management of patients with IHC 2+ and ISH *HER2/CEP17* ratio <2.0; average *HER2* copy number ≥4.0 and <6.0 test results is challenging.

The updated 2018 ASCO/CAP HER2 guidelines address the difficulty in classifying this group of tumors referred to as ISH group 4 (*HER2/CEP17* ratio <2.0; average *HER2* copy number ≥4.0 and <6.0; 2013 ASCO/CAP ISH equivocal). Concurrent IHC testing of the same sample is recommended. If IHC is 3+, the tumors are considered HER2 positive in the 2018 guidelines. If IHC is 0 or 1+, the tumors are considered HER2 negative. If IHC is 2+, additional ISH testing by an observer blinded to the previous results to recount at least 20 cells is recommended. If the result of the recount remains the same (*HER2/CEP17* ratio <2.0; average *HER2* copy number ≥4.0 and <6.0), then the tumor is considered HER2 negative.^9^ While the new guideline represents a much-needed practical approach to these difficult to categorize tumors, more information about the biology of these tumors, and in particular, more data about outcome after trastuzumab treatment in this group of tumors, would help support treatment decisions. Therefore, we compared HER2 mRNA and protein levels in 2018 ASCO/CAP ISH group 4 with IHC 2+ and IHC 0/1+ results on initial biopsy using RT-qPCR and quantitative immunofluorescence (QIF). We also assessed the correlation between RT-qPCR, QIF, conventional IHC, and FISH ratio/copy number. In addition, we gathered information on anti-HER2 treatment, recurrence, and survival in this patient cohort.

## Results

### RT-qPCR and QIF results

RT-qPCR score for all 63 samples ranged from −5.3 to 1.1, average −1.3. Twenty-five samples were classified as positive with RT-qPCR ranging from −1.0 to 1.1 (average −0.5). Thirty-eight samples were classified as negative with RT-qPCR ranging from −5.3 to −1.1 (average −1.9). Supplementary Fig. 1 shows examples of QIF in 2018 ASCO/CAP ISH group 4 breast cancer samples (figshare: 10.6084/m9.figshare.8863583). QIF AQUA score range and average for all 63 samples was 156.9 to 7597.7 and 1757.7, respectively. Of the 63 samples, 18 samples were classified as positive (range 1930.6 to 7597.7 and average 3424.0) and 45 samples were classified as negative (range 156.9 to 1825.3 and average 1091.2) by QIF AQUA score.

### Comparison of IHC versus RT-qPCR and QIF

**Fig. 1:**
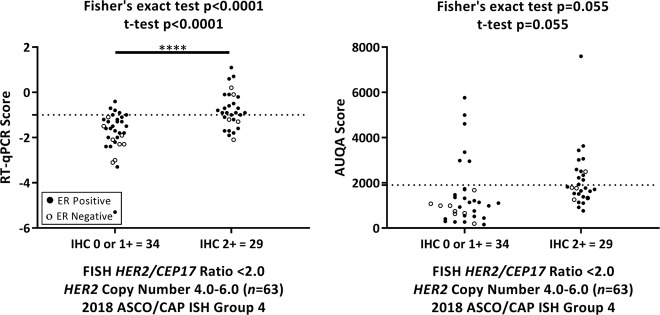
Analysis of human epidermal growth factor receptor 2 immunohistochemistry (IHC) status by real-time quantitative reverse transcription polymerase chain reaction (RT-qPCR) and quantitative immunofluorescence. Distribution of RT-qPCR (**a**) and AQUA (**b**) scores in 2018 American Society of Clinical Oncology/College of American Pathologists in situ hybridization group 4 IHC 0/1+ and IHC 2+ tumors. Closed and open circle represent estrogen receptor-positive and -negative cases, respectively. Dotted line represents the threshold for RT-qPCR or AQUA. Significant *P* values are represented as four asterisks (****) for <0.0001

We determined the HER2 status in initial biopsies of 63 patients with 2018 ASCO/CAP ISH group 4 FISH results (34 with IHC 0/1+ and 29 with IHC 2+) using RT-qPCR and QIF. Among tumors in ASCO/CAP ISH group 4 when compared with IHC 0/1+ tumors, those with IHC 2+ had higher *HER2* mRNA levels (Fisher’s exact test and *t* test *P* < 0.0001) but comparable HER2 protein levels (Fisher’s exact test and *t* test *P* = 0.055). Among 29 IHC 2+ biopsies, RT-qPCR classified 19 (65.5%) as *HER2* positive and the remaining 10 as *HER2* negative, whereas QIF classified 12 (41.3%) as HER2 positive and the remaining 17 as HER2 negative. For the 34 IHC 0/1+ biopsies, both RT-qPCR and QIF classified 6 (17.6%) as HER2 positive and the remaining 28 as HER2 negative (Fig. 1).

### Sensitivity and specificity assessments of RT-qPCR and QIF

**Fig. 2:**
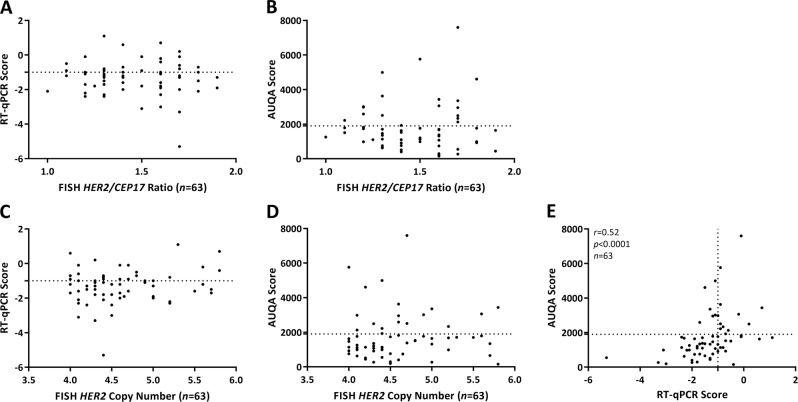
Correlation of human epidermal growth factor receptor 2 (HER2) fluorescence in situ hybridization (FISH) *HER2/CEP17* ratio and *HER2* copy number with real-time quantitative reverse transcription polymerase chain reaction (RT-qPCR) and quantitative immunofluorescence (QIF). Scatter plot distribution between RT-qPCR and FISH *HER2/CEP17* ratio/*HER2* copy number (**a**, **c**), between QIF and FISH *HER2/CEP17* ratio/*HER2* copy number (**b**, **d**), and between RT-qPCR and QIF (**e**). Dotted line represents the threshold for RT-qPCR or AQUA

By both RT-qPCR and QIF, 12 specimens were HER2 positive and 32 specimens were HER2 negative (Supplementary Table 1). Of the 63 specimens with ASCO/CAP ISH group 4 FISH results, concordance agreement for positive and negative between RT-qPCR and QIF was 69.8% (*P* = 0.0096, sensitivity = 0.48; specificity = 0.84; positive predictive value = 0.70; negative predictive value = 0.71, Supplementary Table 1) and the correlation was Spearman *r* = 0.52 (*P* < 0.0001, Fig. 2e).

### Distribution of RT-qPCR and QIF results versus FISH ratio/copy number

We also compared the performance of the RT-qPCR and QIF methods relative to the FISH *HER2/CEP17* ratio and *HER2* copy number in 63 specimens with ASCO/CAP ISH group 4 FISH results. The distribution results of RT-qPCR and AQUA scores were both independent of FISH *HER2/CEP17* ratio (Fig. 2a, b). Similar results were obtained when RT-qPCR and AQUA scores were plotted against *HER2* copy number (Fig. 2c, d).

### Results of repeat testing

**Fig. 3:**
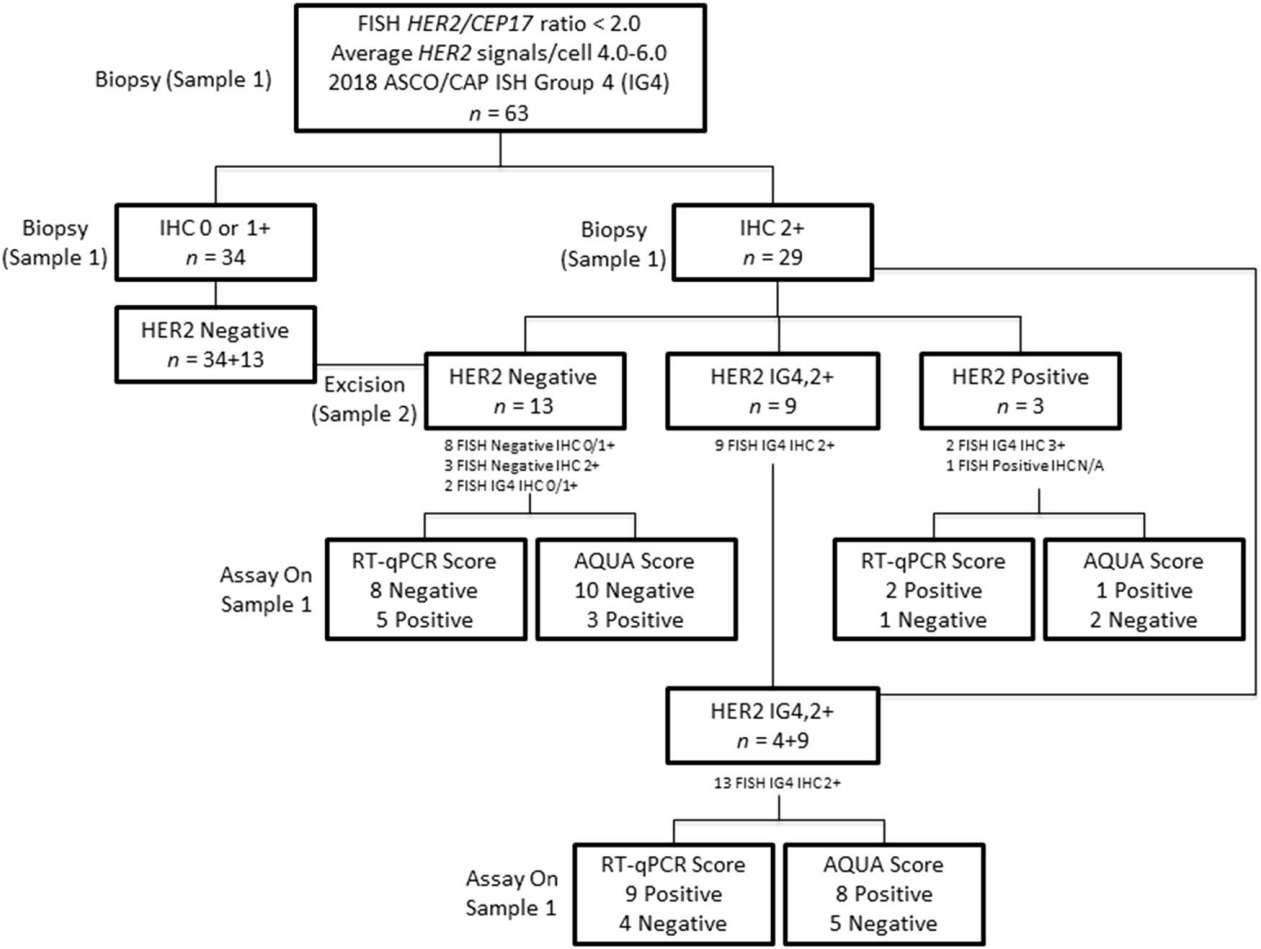
Flowchart identifying potentially treatable patients using real-time quantitative reverse transcription polymerase chain reaction (RT-qPCR) and quantitative immunofluorescence (QIF). Biopsy (sample 1) is initial specimen and excision (sample 2) is alternative specimen from same patient. RT-qPCR and QIF assays were performed on biopsy (sample 1), the initial specimen. IG4,2+: 2018 American Society of Clinical Oncology/College of American Pathologists in situ hybridization group 4 and IHC 2+

Among the 63 patients with ASCO/CAP ISH group 4 FISH results, the HER2 IHC result of the initial biopsy (sample 1) was IHC 2+ for 29 and IHC 0/1+ for 34 patients. Of these 29 patients with ASCO/CAP ISH group 4 FISH results and IHC 2+ results on the initial biopsy, repeat testing on the excision (sample 2) reduced the number of ASCO/CAP ISH group 4 FISH results and IHC 2+ results by >50% (16 cases) and classified them into either HER2 positive (*n* = 3) or HER2 negative (*n* = 13). The remaining 13 samples remained as ASCO/CAP ISH group 4 FISH results and IHC 2+ (Fig. 3). Thus, after repeat testing, the HER2 status for the 63 patients was 47 (74.6%) HER2 negative, 13 (20.6%) ASCO/CAP ISH group 4 FISH results and IHC 2+, and 3 (4.8%) HER2 positive.

### Adjudication of HER2 status using RT-qPCR and QIF

Among the 16 patients whose HER2 results were adjudicated into HER2 negative or positive by repeat testing on the excision, the results of RT-qPCR and QIF agreed in 10 (62.5%) and 11 (68.8%) of the patients, respectively (Fig. 3). Among the 13 patients for whom HER2 results remained ASCO/CAP ISH group 4 and IHC 2+ after repeat testing, RT-qPCR classified 9 tumors as *HER2* positive and the remaining 4 tumors as *HER2* negative on the biopsy. On the other hand, QIF classified 8 tumors as HER2 positive on biopsy, but only 8 cases agreed with the RT-qPCR classification (Fig. 3).

### Systemic treatment and outcome

**Fig. 4:**
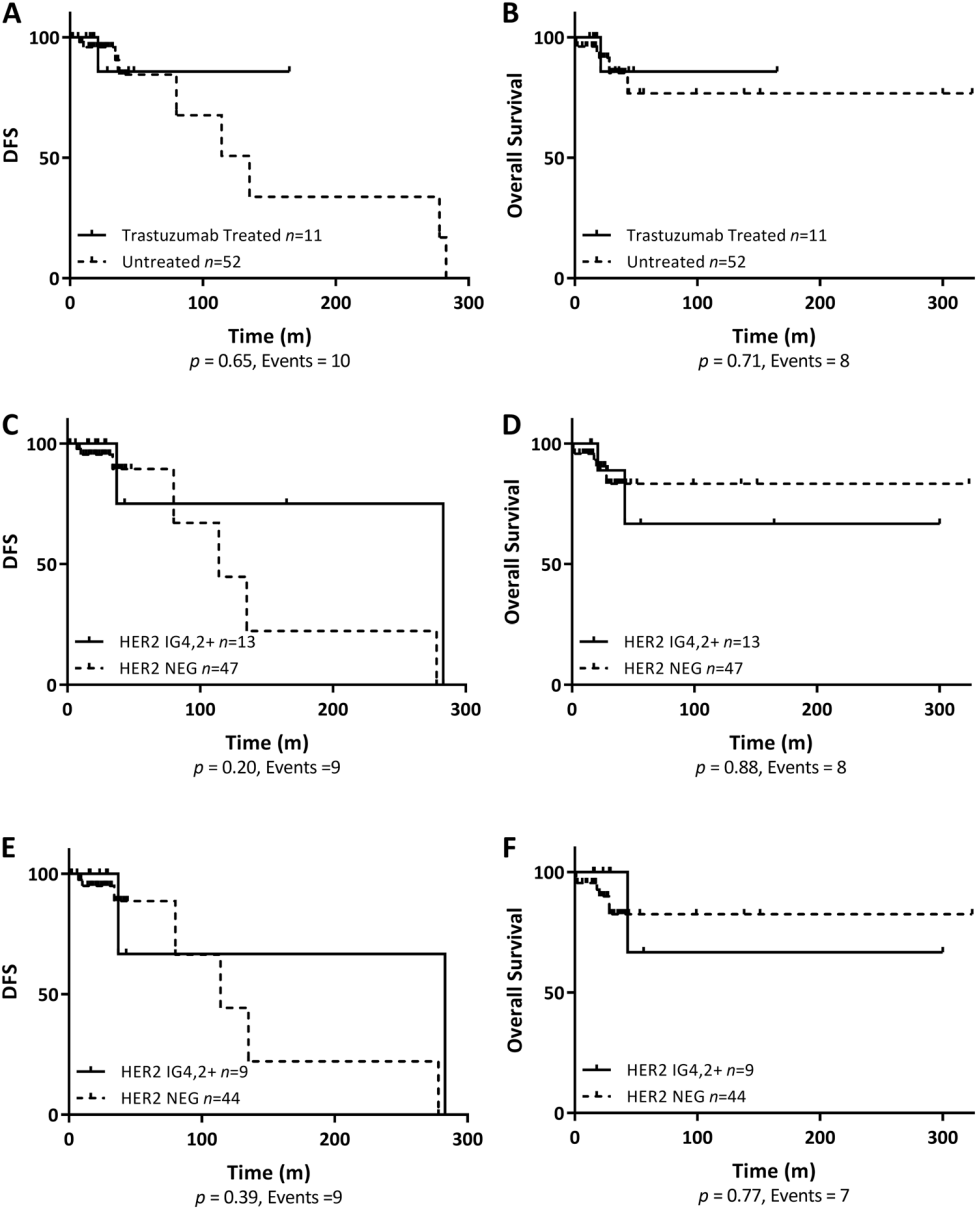
Survival according to trastuzumab treatment and human epidermal growth factor receptor 2 (HER2) status. Kaplan–Meier graphical analysis of DFS and OS according to trastuzumab treatment status (**a**, **b**), HER2 status (**c**, **d**) and HER2 status excluding trastuzumab treated patients (**e**, **f**). IG4,2+ 2018 American Society of Clinical Oncology/College of American Pathologists in situ hybridization group 4 and IHC 2+, NEG negative

Among the 63 patients with ASCO/CAP ISH group 4 HER2 FISH results, 33 (52.4%) patients received chemotherapy, 45 (71.4%) patients received hormonal therapy, and 11 (17.5%) patients received trastuzumab. No statistically significant difference in disease-free survival (DFS) and overall survival (OS) was observed for trastuzumab-treated patients versus untreated patients (Fig. 4a, b).

We also correlated RT-qPCR and QIF results with treatment and outcome data. Of the 11 trastuzumab-treated patients, RT-qPCR classified the tumors of 4 patients as *HER2* positive with 1 recurrence and no death and the tumors of the remaining 7 patients as *HER2* negative with 1 death and no recurrence. Among the 52 trastuzumab untreated patients, RT-qPCR classified the tumors of 21 as *HER2* positive with 5 events (4 recurrences and 1 death) and the tumor of the remaining 31 as *HER2* negative with 11 events (6 recurrences and 5 deaths). Similar trends were observed for QIF (Supplementary Table 2).

Systemic treatment was compared between patients with HER2 negative and ASCO/CAP ISH group 4 and IHC 2+ status. No statistically significant difference was observed in terms of chemotherapy (*P* = 0.53), hormonal therapy (*P* = 0.48), radiation therapy (*P* = 0.22), and trastuzumab treatment (*P* = 0.06) (Table 1). No specific correlation was observed between estrogen receptor (ER) status and trastuzumab treatment in this patient cohort (*P* = 0.70, Supplementary Table 3).

**Table 1:** Summary of outcome data for HER2 negative and HER2 2018 ASCO/CAP ISH group 4 and IHC2+ (IG4,2+) status patient cohort

Characteristics	HER2 negative, *n* = 47 (%)	HER2 IG4,2+, *n* = 13 (%)	*P* value
Treatment			
Chemotherapy	22 (46)	8 (61)	0.53
Hormonotherapy	33 (70)	11 (85)	0.48
Radiotherapy	28 (60)	5 (38)	0.22
Trastuzumab	4 (9)	4 (31)	0.06
Recurrence			1.0
Yes	7 (15)	2 (15)	
No	40 (85)	11 (85)	
Survival			1.0
Deceased	6 (13)	2 (15)	
Alive	41 (87)	11 (85)	

All statistical tests were two-sided Fisher’s exact test

*ASCO/CAP* American Society of Clinical Oncology/College of American Pathologists, *HER2* human epidermal growth factor receptor 2, *ISH* in situ hybridization

In terms of clinical outcome, no statistically significant difference was observed between patients with HER2 negative and HER2 ASCO/CAP ISH group 4 and IHC 2+ status for DFS and OS (Fig. 4c, d). Furthermore, when trastuzumab-treated patients were excluded from HER2 negative and HER2 ASCO/CAP ISH group 4 and IHC 2+ cohorts, no difference was observed in DFS and OS (Fig. 4e, f).

## Discussion

The 2018 ASCO/CAP HER2 testing in breast cancer clinical practice guideline focused update recommends a much needed practical approach to difficult situations where *HER2* ISH results are difficult to classify, such as for breast cancers with *HER2/CEP17* ratio <2.0; average *HER2* copy number ≥4.0 and <6.0, referred to as 2018 ASCO/CAP ISH group 4 and 2013 ASCO/CAP ISH equivocal.^9^ Little information about prognosis and response to anti-HER2 treatment is available for this subset of patients. The question remains if this subset of patients represents a distinct biological group. Here we used two quantitative methods, RT-qPCR and QIF, to evaluate HER2 mRNA and protein levels in 2018 ASCO/CAP ISH group 4 breast cancers. Both quantitative methods provide continuous values without an equivocal category and have potential for more accurate assessment of HER2 in a timely manner.

Alternative assays including RT-qPCR, FISH with alternative control probes for chromosome 17, and genomic profiling have been suggested to resolve HER2 ambiguity in 2018 ASCO/CAP ISH group 4 breast cancers.^3,5–8,10^ Our study included trastuzumab-treated 2018 ASCO/CAP ISH group 4 breast cancers and we attempted to assess systemic treatment patterns and disease outcome.

In our study, among 2018 ASCO/CAP ISH group 4 breast cancers, RT-qPCR scores are higher in those with IHC 2+ but AQUA scores are similar in those with IHC 2+ compared to those with IHC 0/1+. This discrepancy between two quantitative methods could be because total mRNA does not reflect total protein and vice versa. Other studies using previous guidelines and different patient selection showed different findings: Wang et al. reported no significant difference in RT-qPCR *HER2* score between FISH equivocal IHC 2+ versus FISH equivocal IHC 0/1+ tumors as defined by the 2007 ASCO/CAP guidelines rather than the 2013 ASCO/CAP guidelines;^5^ Tong et al. found comparable RT-qPCR expression levels for FISH equivocal IHC 2+ and FISH negative IHC 2+ tumors, which is a different population (all IHC 2+ rather than all FISH equivocal) than our study;^10^ and Marchio et al. found significantly different *HER2* mRNA levels between HER2 positive, HER2 negative, and double equivocal carcinomas (HER2 ISH group 4 and IHC 2+ carcinomas) with greater overlap of *HER2* mRNA levels between HER2 double equivocal and HER2 negative carcinomas.^8^ ISH group 4 tumors represent the center in the continuum of *HER2* expression.

Correlation between RT-qPCR and QIF methods was poor in our 2018 ASCO/CAP ISH group 4 cohort for cases with IHC 2+ but was excellent (80%) for cases with IHC 0/1+. Interestingly, each method misclassified six HER2 negative cases as positive and the two misclassified cases were concordant between these two methods. The cases misclassified by RT-qPCR were close to the cutoff for *HER2*, which might be responsible for the different result by this non-conventional assay. Our data over the past 2 years supports higher concordance (94%) between these methods for unselected breast cancer samples.^11–13^ In this study of 2018 ASCO/CAP ISH group 4 breast cancers that are difficult to classify by conventional testing, most samples also showed RT-qPCR and QIF results close to positive/negative cut points. In contrast in breast carcinomas without equivocal results by conventional testing, both RT-qPCR and QIF showed a wider range in scores further from positive/negative cut points. The range and average RT-qPCR score in a series of unselected breast carcinomas was −10.1 to 2.3 and −2.0, with 0.6 the average score for carcinomas classified as positive by RT-qPCR. The range and average QIF AQUA score in a series of unselected breast carcinomas was 135.5 to 15087.5 and 3282.8, with 7281.3 the average score for carcinomas classified as positive by QIF.^12^ By chance due to random errors associated with any test results close to the cut points could be considered false positive or false negative results compared to conventional testing. Adjudication of these results into negative or positive by repeat testing with multiple methods or different arbitrary cut points is unlikely to be meaningful. Additional outcome information after trastuzumab treatment for patients with tumors with difficult-to-classify HER2 results will shed light on how best to classify these tumors. Furthermore, the RT-qPCR and AQUA scores of our 2018 ASCO/CAP ISH group 4 specimens were independent of FISH *HER2/CEP17* ratio and average *HER2* copy number. Similarly, Tong et al. reported that RT-qPCR scores of *HER2* FISH equivocal tumors were independent of FISH *HER2/CEP17* ratio and *HER2* copy number,^10^ whereas in *HER2* unequivocal breast cancer, tight correlation between RT-qPCR and FISH ratio/copy number is seen.^5^

No statistically significant difference was observed for chemotherapy, endocrine therapy, radiation therapy, and anti-HER2 targeted therapy between patients with HER2 negative and HER2 ASCO/CAP ISH group 4 and IHC 2+ status. Eleven patients with *HER2* ASCO/CAP ISH group 4 breast cancers were treated with anti-HER2 targeted therapy, trastuzumab. Per ASCO/CAP 2013 guidelines, anti-HER2 targeted therapy may be considered in patients with equivocal IHC and ISH results. We attempted to find out what influenced the choice of trastuzumab treatment for these patients. Possible reasons include: (1) ISH- or IHC-positive results on a subsequent specimen; (2) oncologist recommended chemotherapy and trastuzumab with trastuzumab added because the patient would be given chemotherapy, whereas trastuzumab may not have been recommended if chemotherapy was decided against; or (3) stage IV metastatic disease. Only one trastuzumab-treated patient was stage IV. No correlation was found between factors such as grade, ER status, and progesterone receptor (PR) status, which influence the decision to give chemotherapy and trastuzumab treatment. Only 2 (18.1%) recurrences and/or deaths were observed in trastuzumab-treated patients with 2018 ASCO/CAP ISH group 4 breast cancers and the remaining 16 (28.8%) recurrences and/or deaths were observed in patients with 2018 ASCO/CAP ISH group 4 breast cancers who did not receive trastuzumab. Although not statistically significant, a slightly better DFS and OS was observed for trastuzumab-treated patients versus untreated patients in our study. Interestingly, in 10 double equivocal (ISH group 4 and IHC2+ carcinomas) treated with neoadjuvant chemotherapy and trastuzumab, Marchio et al. reported a statistically significant lower pathologic complete response (pCR) rate. But this statistically different response rate faded away when carcinomas with pCR and with minimal residual disease, near total effect, or <10% of tumor remaining were grouped and compared to ER- and Ki67-matched HER2 IHC 3+ carcinomas. Recurrence or survival information was not available in their cohort.^8^ We found overlapping DFS and OS curves for patients with HER2 IHC 2+ and HER2 IHC 0/1+ results among patients with 2018 ASCO/CAP ISH group 4 breast cancer. Several other studies also found similar survival outcomes between these two subsets.^7,14^

Our ability to correlate outcome with HER2 results and trastuzumab treatment was limited by small numbers. The 2018 ASCO/CAP ISH group 4 results were seen in 5% (94 of 1862) of breast cancer specimens tested at Yale University. A rate similar to the rate reported in the literature was observed. We could not associate outcomes (recurrence and/or death) to adjudication of HER2 status in our cohort of 2018 ASCO/CAP ISH group 4 by RT-qPCR and QIF.^5^ Only 17.4% (11 of 63) patients with 2018 ASCO/CAP ISH group 4 HER2 results were treated with trastuzumab, further reducing the number of patients/events in the trastuzumab-treated cohort. Marchio et al. identified 4% (2 of 45) ISH group 4 and IHC2+ carcinomas as HER2-enriched subtype by the Prosigna assay.^8^ This observation together with our limited observations on survival and recurrence in trastuzumab-treated patients and Marchio et al.’s preliminary observation on pathologic response after neoadjuvant trastuzumab suggests that even though these tumors are rare the possibility of benefit from anti-HER2 treatment in some of these tumors is worth investigating. Another limitation of our study is that HER2 IHC and FISH results were collected from the pathology reports before reporting of heterogeneity was standardized at our institution. The STRAT4 assay does not detect heterogeneity as it is performed from a macrodissected tissue lysate. Furthermore, we averaged HER2 expression by QIF across the tumor area. Therefore, we were unable to examine the role of intratumor *HER2* genetic heterogeneity.

In conclusion, levels of RNA and protein expression in 2018 ASCO/CAP ISH group 4 tumors were intermediate, close to positive/negative cut points. In other words, few 2018 ASCO/CAP ISH group 4 tumors show RNA or protein expression clearly negative or positive far away from cut points. Systemic treatment, recurrence, and survival outcomes were comparable among ISH group 4 cancers regardless of IHC 0/1+ or 2+ results. Together these observations suggest that 2018 ASCO/CAP ISH group 4 tumors are a true biological subset, representing the continuum of *HER2* expression in breast cancer. The inability to classify these cases, by any of the four methods tested, illustrates the problem of finding a definitive cut point in a continuous population. Adjudication into negative or positive categories by chance may not be meaningful. Our findings support the 2018 ASCO/CAP recommendation to refrain from routine additional testing of these samples. Because 2018 ASCO/CAP ISH group 4 tumors appear to be similar to HER2 negative tumors in terms of prognosis, the practical approach recommended by the ASCO/CAP guidelines makes sense, particularly considering the results of the NSABP-47 trial, which found no benefit of trastuzumab in patients with IHC 1+ or 2+ and negative ISH.^15^ However, this trial did not include patients with ASCO/CAP 2018 group 4 ISH results. Additional outcome information after trastuzumab treatment for patients in this special group might help inform treatment decisions in these patients.

## Methods

### Patients and tissue samples

Patients were selected by searching the Yale Pathology electronic database for samples of invasive breast carcinoma with HER2 FISH *HER2/CEP17* ratio <2.0; average *HER2* copy number ≥4.0 and <6.0 results from 2014 to 2017. A total of 63 patients were included. Of the 63 patients, 29 patients had IHC 2+ and 34 patients had IHC 0/1+ results on initial biopsy. Among the 63 patients after repeat biopsies, 47 (74.6%) were HER2 negative, 13 (20.6%) were HER2 2+ and 2018 ASCO/CAP ISH group 4, and 3 (4.8%) were HER2 positive. A summary of patient clinicopathologic characteristics is shown in Table 2. HER2 RT-qPCR and QIF studies were performed on initial biopsies obtained from the 63 patients. The results for Clinical Laboratory Improvement Amendments-certified HER2 IHC and FISH scoring according to the ASCO/CAP 2013 guidelines were extracted from the pathology reports^1^. This study complies with all ethical regulations and is approved by IRB protocol ID 9505008219, Yale University, with the waiver of informed consent.

**Table 2:** Summary of patient clinicopathologic characteristics

Characteristics	*n* = 63 (%)
Age (years)	
Range	27–94
Mean	68
TNM stage	
I	26 (41)
II	25 (40)
III	7 (11)
IV	5 (8)
Histological type	
Ductal	43 (68)
Ductal/micropapillary	12 (19)
Ductal/lobular	5 (8)
Other	3 (5)
Histological grade	
I	5 (8)
II	37 (59)
III	21 (33)
Molecular markers	
ER positive	50 (79)
PR positive	42 (67)

Histological type other (3) includes 1 lobular, 1 micropapillary and 1 squamous

*ER* estrogen receptor, *PR* progesterone receptor, *TNM* Tumor, Node, Metastasis

### Quantitative RT-PCR

The Breast Cancer STRAT4 Research Use Only (RUO) assay was performed as previously described.^11,12^ STRAT4 is a CE-IVD (Conformité Européene In-vitro Medical Device) product that is available in some, but not all, European countries and is not available in the United States. Where the STRAT4 assay is not available under CEIVD, evaluations of its performance using specimens prepared under local pre-analytical sample handling procedures can be supported under collaborative research agreements using a RUO version. Briefly, 5-µM thick FFPE (formalin-fixed paraffin-embedded) tissue sections were collected and macrodissected to collect tumor. Samples were mixed with 20 µL Proteinase K and 1.2 mL FFPE lysis reagent (Cepheid). After a 30-min incubation at 80 °C, 1.2 mL of >95% ethanol was added to the lysed samples and vortexed to mix. Five hundred and twenty microliters of this mixture was then transferred to the cartridge and run on the GX system. This assay isolates the total RNA, performs a 1-step RT-PCR, and provides Ct values for both the *CYFIP1* endogenous control and four target genes, including the *ERBB2* transcript using proprietary primer sequences. Results are expressed as a delta cycle threshold (dCt) value, defined as the Ct of the control gene, *CYFIP1*, minus the Ct of the target gene. The BC STRAT4 dCt cutoff for *ERBB2* assigns two categories (positive and negative) and was validated in multiple breast cancer patient cohorts, based on the highest concordance achieved between relative RNA amplification and corresponding protein expression by IHC, where FISH was performed in all IHC 2+ cases.

### Quantitative immunofluorescence

FFPE sections were deparaffinized at 60 °C for at least 30 min, then incubated twice in xylene for 20 min. Rehydration was done using ethanol. Antigen retrieval was performed as recommended by the manufacturer’s protocol with citrate buffer pH 6.0 at 97 °C for 20 min in a pressure-boiling container (Lab Vision, PT Module, Thermo Fischer Scientific, Waltham, MA). Endogenous peroxidase activity was blocked with 2.5% hydroxyl peroxide in methanol for 30 min, followed by blocking with 0.3% bovine serum albumin in 0.1 mol/L of Tris-buffered saline for 30 min at room temperature. The commercially available primary mouse monoclonal anti-HER2 antibody (CB11, Biocare Medical, Concord, CA, USA) was used at a concentration of 1:625 or 1:800.^16^ Sections were then incubated overnight at 4 °C with the primary antibody and cytokeratin at 1:100 dilution (polyclonal rabbit anti-cytokeratin, wide spectrum screening, Agilent Technologies, Santa Clara, CA, USA). Slides were incubated for an hour at room temperature with Alexa 546-conjugated goat anti-rabbit secondary antibody (Thermo Fisher Scientific, Waltham, MA, USA) at 1:100 dilution in mouse EnVision amplification reagent (Dako). Cyanine 5 directly conjugated to tyramide (Perkin-Elmer, Waltham, MA, USA) at 1:50 dilution was used for target antibody detection. ProLong mounting medium (ProLong Gold; Molecular Probes) with 4,6-diamidino-2-phenyl-indole was used to stain nuclei.

### Fluorescence measurement and scoring

QIF staining was performed using the AQUA method as previously reported by our group.^16–18^ Briefly, the HER2 QIF score in the tumor compartment was calculated by dividing the sum of the HER2 compartment pixel intensities by the area of cytokeratin positivity resulting in a continuous score directly proportional to the concentration of the biomarker of interest. QIF scores were normalized to the exposure time and bit depth at which the images were captured, allowing scores collected at different exposure times to be comparable. All acquired histospots were visually evaluated and cases with staining artifacts or <2% tumor determined by cytokeratin staining were excluded from the analysis. Index tissue microarrays (TMAs) were used to standardize all autostainer runs and to define the QIF score cut point that is equivalent to the clinical cut point for the index TMA cases.

### Statistical analysis

We extracted the information from the 63 patient cohort. The comparison among 2018 ASCO/CAP ISH group 4 with IHC 2+ and IHC 0/1+ results was performed using two-sided Fisher’s exact and two-tailed unpaired Student’s *t* test. Spearman correlation was computed for RT-qPCR and QIF tests. Two-sided Fisher’s exact test was also used to compare concordance between the RT-qPCR, IHC, and QIF results. DFS and OS were compared using Kaplan–Meier estimates, and statistical significance was determined using log-rank test. All data sets were analyzed and plotted using the GraphPad Prism v7.0 software for Windows (GraphPad Software, Inc., La Jolla, CA). Significant *P* values are represented as four asterisks (****) for <0.0001.

### Reporting summary

Further information on research design is available in the Nature Research Reporting Summary linked to this article.

## Supplementary information

Supplementary Information

Reporting Summary Checklist

## Data Availability

The data generated and analyzed during this study are available from the corresponding author on reasonable request. Data file accessibility and description of data files are provided in the following metadata record: 10.6084/m9.figshare.8863583.^19^ Quantitative immunofluorescence images are publicly available in Supplementary Fig. 1 of the published article.

## Supplementary information

**Supplementary information** accompanies the paper on the *npj Breast Cancer* website (10.1038/s41523-019-0122-x).
