# Supplementary material for: Quantitative assessments and clinical outcomes in HER2 equivocal 2018 ASCO/CAP ISH group 4 breast cancer
Source: NPJ Breast Cancer. 2019 Aug 29;5:28. doi: 10.1038/s41523-019-0122-x (PMC6715641; doi:10.1038/s41523-019-0122-x)
Supplement: Supplementary file 1 — Supplementary Information [file 41523_2019_122_MOESM1_ESM.pdf]

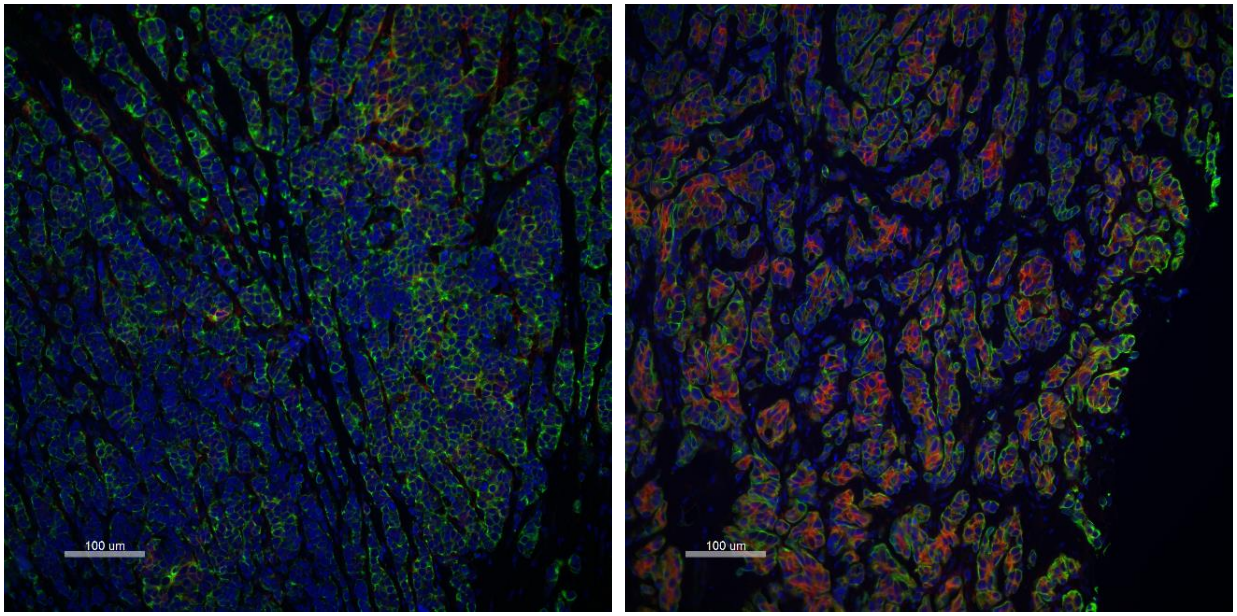

Supplementary Figure 1. Representative image of a 2018 ASCO/CAP ISH group 4 breast carcinoma that is negative (left) and positive (right) by QIF. Blue = DAPI, Green = Cytokeratin and Red = HER2.

Supplementary Table 1: Concordance between RT-qPCR and QIF.

	dCt ≥-1	dCt <-1	Total
≥ 1900 AU	12	6	18
< 1900 AU	13	32	45
Total	25	38	63

P = 0.0096; sensitivity = 0.48; specificity = 0.84; PPV = 0.67; NPV = 0.71)

Supplementary Table 2: Assessment of RT-qPCR and QIF results using outcome data.

RT-qPCR	Trastuzumab	RT-qPCR Score	Recurrence (*n*)	Death (*n*)
	Treated, *n* = 11	Positive, *n* = 4	1	0
		Negative, *n* = 7	0	1
	Untreated, *n* = 52	Positive, *n* = 21	4	1
		Negative, *n* = 31	5	6
QIF	Trastuzumab	AQUA Score	Recurrence (*n*)	Death (*n*)
	Treated, *n* = 11	Positive, *n* = 4	1	0
		Negative, *n* = 7	0	1
	Untreated, *n* = 52	Positive, *n* = 14	1	2
		Negative, *n* = 38	8	5

Supplementary Table 3: Comparison of outcome data between ER positive and negative patients.

**Characteristics**	**ER Positive** *n* **= 50 (%)**	**ER Negative** *n* **= 13 (%)**	***P* value**
Treatment			
Chemotherapy	25 (51)	8 (62)	.54
Hormonotherapy	44 (88)	1 (8)	<0.0001***
Radiotherapy	26 (53)	8 (62)	.76
Trastuzumab	8 (18)	3 (38)	.70
Recurrence			1.0
Yes	8 (16)	2 (15)	
No	42 (84)	11 (85)	
Survival			.66
Deceased	6 (12)	2 (15)	
Alive	44 (88)	11 (85)	

All statistical tests were two-sided fisher’s exact test.
